# Comprehensive radiological and clinical evaluation of coarctation of the aorta with bicuspid aortic valve and ascending aortic aneurysm: A case report

**DOI:** 10.1016/j.radcr.2025.03.077

**Published:** 2025-04-24

**Authors:** Hafsi Azer, Hafsi Fares, Ganzoui Imen

**Affiliations:** Department of Radiology, Habib Bougatfa Hospital, Bizerte, Tunisia

**Keywords:** Coarctation of the aorta, Bicuspid aortic valve, Ascending aortic aneurysm, Computed tomography angiography, Collateral circulation, Multidisciplinary management

## Abstract

Coarctation of the aorta (CoA) and bicuspid aortic valve (BAV) disease are frequently coexisting congenital anomalies that pose significant diagnostic and therapeutic challenges. This report highlights a rare case of severe CoA with aneurysmal dilation of the ascending aorta in a 31-year-old patient. The patient underwent advanced imaging with synchronized aortic CT angiography to assess the extent of the aortic pathology and associated vascular anomalies. Imaging revealed severe CoA, significant collateral circulation, and an aneurysmal ascending aorta. Additionally, coronary artery anomalies and left ventricular hypertrophy were identified, necessitating a multidisciplinary management approach. This case underscores the importance of comprehensive imaging in the evaluation of complex aortic diseases. Detailed visualization guided the surgical planning for this patient, emphasizing the critical role of advanced imaging modalities in improving patient outcomes.

## Introduction

Congenital heart diseases involving the aorta, such as bicuspid aortic valve (BAV) disease and coarctation of the aorta (CoA), are associated with significant morbidity and mortality if untreated. These conditions can coexist, further complicating the clinical course and management. BAV is the most common congenital heart defect, affecting approximately 1%-2% of the population, and is frequently associated with aortic aneurysms and CoA [1]. This report discusses a rare case of severe CoA associated with an aneurysmal ascending aorta in a young adult, emphasizing the role of advanced imaging in diagnosis and treatment planning.

## Case report

A 31-year-old male with no significant medical history was followed for an ascending aortic aneurysm associated with bicuspid aortic valve (BAV) disease. He presented to the hospital with a hypertensive crisis and left ventricular dysfunction. Laboratory findings were unremarkable.

## Imaging findings

Computed tomography angiography (CTA) was performed, revealing severe coarctation of the aorta, a narrowed segment measuring 8 mm distal to the left subclavian artery origin, and an atretic segment 8 mm in length with pre- and poststricture diameters of 18 mm and 21 mm, respectively. Additionally, a hypodense linear structure, measuring 11 mm, was consistent with a residual ductal remnant extending toward the left pulmonary artery.

**Fig. 1:**
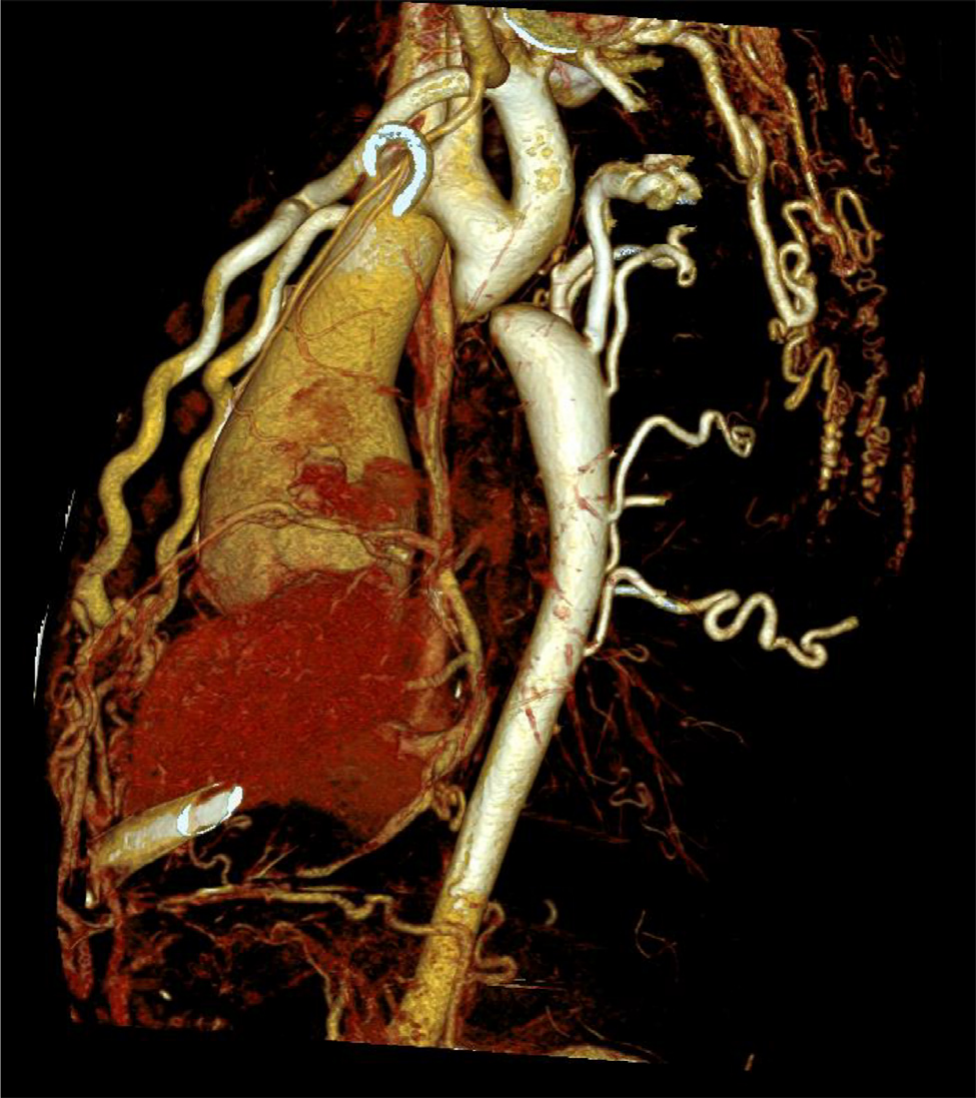
Virtual reconstruction CT scan image demonstrating the coarctation site.

**Fig. 2:**
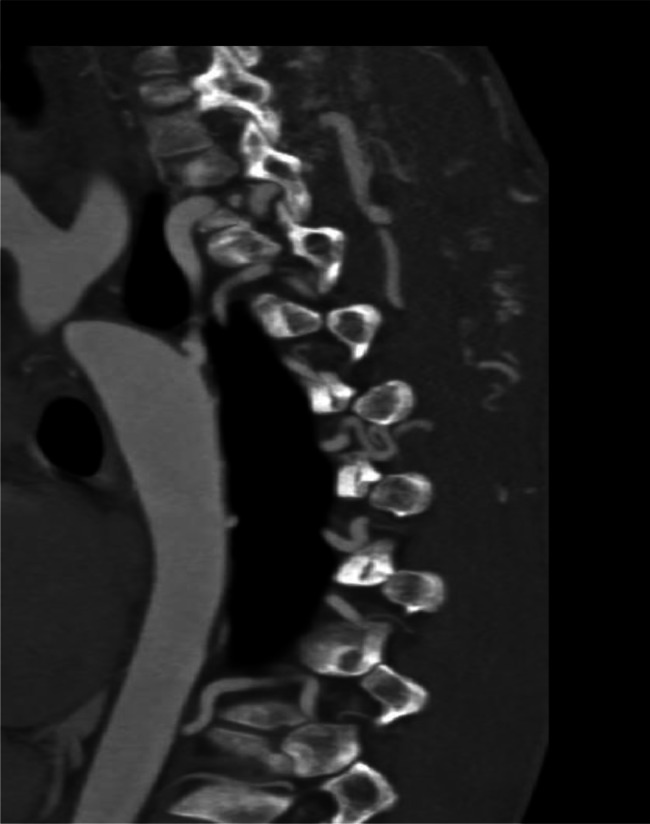
Sagittal reconstruction with MIP reforming showing the hypertrophied collateral vessels.

**Fig. 3:**
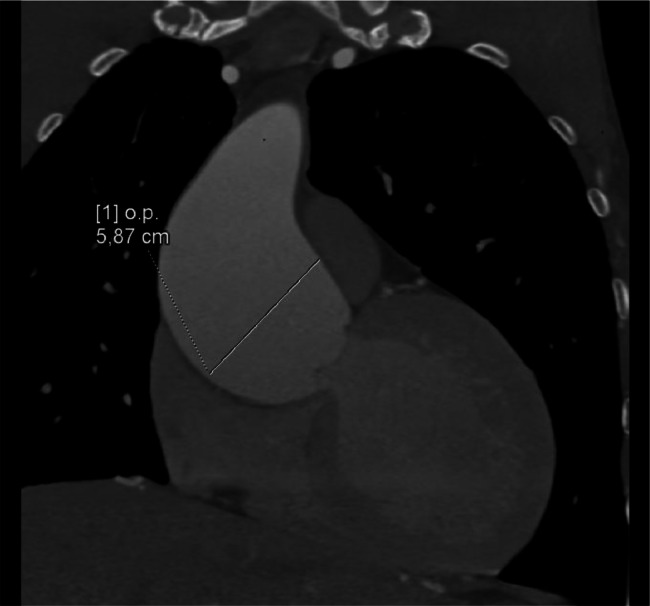
Coronal reconstruction CT image highlighting the aneurysmal ascending aorta.

**Fig. 4:**
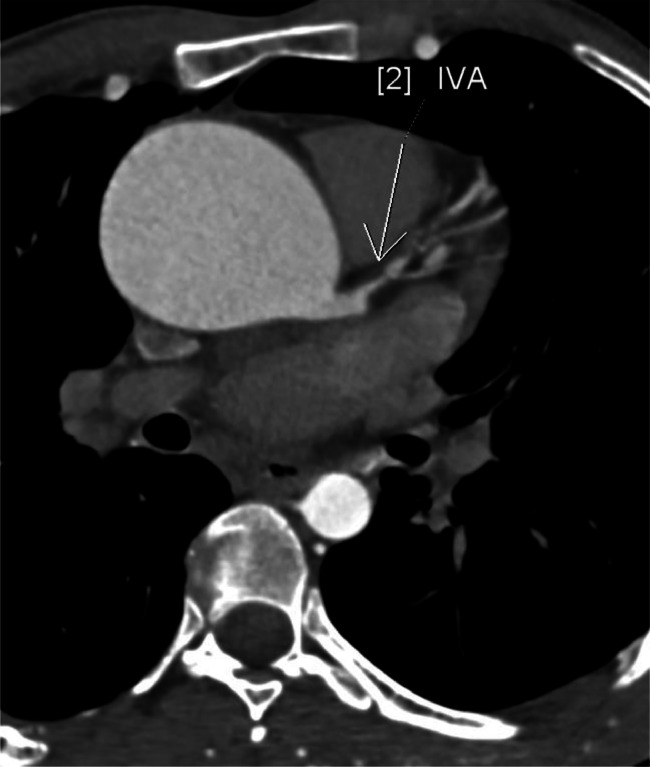
Oblique reconstruction CT image of the first segment of the IVA stenosis.

Extensive collateral circulation was observed, including an aorto-aortic network involving the internal mammary, lateral thoracic, and posterior intercostal arteries. Rib notching was present from the second to the ninth ribs. The ascending aorta exhibited aneurysmal dilation, measuring 58 mm in diameter, with a bulbous morphology characteristic of aneurysmal changes. The bicuspid aortic valve was classified as type LR with a visible raphe and no calcifications. Coronary anomalies included a left-dominant system with stenosis in the left anterior descending and circumflex arteries, along with mild hypoplasia of the right coronary artery. Additionally, left ventricular dilation and concentric hypertrophy of the myocardium were noted (Fig. 1, Fig. 2, Fig. 3, Fig. 4).

## Management plan

The patient was referred for surgical correction of the coarctation and replacement of the aneurysmal ascending aorta. Preoperative coronary angiography was planned to address the identified coronary stenoses. The patient is currently undergoing preoperative anesthesiology preparation. The planned surgery has not yet been performed.

## Discussion

The coexistence of coarctation of the aorta (CoA), bicuspid aortic valve (BAV), and ascending aortic aneurysm presents a multifaceted clinical challenge that underscores the importance of advanced imaging for diagnosis and management. CoA and BAV are often associated due to their shared embryological origins, and the combination of these conditions heightens the risk of progressive aortopathy [2]. This patient's case demonstrates the value of detailed imaging in understanding the full scope of cardiovascular pathology, including coarctation severity, collateral circulation, and associated structural abnormalities [3].

In this patient, synchronized computed tomography angiography (CTA) revealed a severe coarctation of the aorta with a narrowed segment 8 mm distal to the left subclavian artery and a hypodense linear remnant consistent with a ductus arteriosus. The imaging also identified significant collateral circulation involving hypertrophied and tortuous vessels, including internal mammary and lateral thoracic arteries, which bypassed the stenotic segment [4]. These findings are indicative of long-standing CoA, even in an otherwise asymptomatic individual. Collateral circulation can serve as a compensatory mechanism but also adds complexity to surgical planning, as disruption of major collateral pathways can lead to hemodynamic instability [5].

The most striking imaging finding was the aneurysmal dilation of the ascending aorta, measuring 58 mm in diameter with a bulbous morphology. This aortic dilation is characteristic of BAV-related aortopathy, which is driven by intrinsic abnormalities in the aortic wall, such as defective extracellular matrix remodeling [6]. The presence of CoA likely exacerbated this pathology due to increased hemodynamic stress. The patient's aortic dimensions exceeded the surgical threshold for intervention, highlighting the importance of timely diagnosis and management in preventing life-threatening complications such as rupture or dissection [7].

The CTA also provided valuable information on the morphology of the bicuspid aortic valve, which was classified as type LR with a visible raphe and no calcifications. This is clinically significant, as the lack of valvular calcification suggests that surgical intervention can focus on repairing the coarctation and aneurysm without immediate need for valve replacement [8]. However, the coronary artery findings—including moderate stenoses in the left anterior inter ventricular and circumflex arteries—warrant preoperative coronary angiography to guide potential concurrent interventions during surgery [9].

Radiological imaging also elucidated additional structural features, such as concentric left ventricular hypertrophy and normal pulmonary venous return, which further informed the clinical picture [10]. The left ventricular hypertrophy is consistent with long-standing pressure overload due to CoA. Pulmonary venous anatomy was unremarkable, ruling out additional congenital abnormalities that could complicate surgical repair [11].

This case highlights the indispensable role of advanced imaging modalities such as CTA in the assessment and management of complex aortic pathologies. The detailed anatomical and functional information obtained through imaging not only confirmed the diagnosis but also facilitated precise surgical planning [12]. Collaboration between radiologists, cardiologists, and cardiovascular surgeons is essential to optimize patient outcomes, particularly in cases where multiple congenital and acquired anomalies coexist. Long-term follow-up with periodic imaging will be critical for this patient to monitor for potential complications such as recoarctation, aneurysm progression, or valve dysfunction after surgical repair [13].

## Conclusion

This case underscores the critical importance of advance D-Imaging in the evaluation and management of complex cardiovascular anomalies such as coarctation of the aorta, bicuspid aortic valve, and ascending aortic aneurysm. Synchronized computed tomography angiography provided comprehensive anatomical and functional details, guiding surgical planning and highlighting the necessity of multidisciplinary collaboration. Timely diagnosis and intervention are essential to mitigate risks of life-threatening complications. Long-term surveillance is pivotal in ensuring optimal patient outcomes.

## Patient consent

In accordance with ethical standards and institutional requirements, informed consent was obtained from our patient included in this study. The patient was fully informed about the purpose of the study, the nature of the images, and how their data would be used for research and publication. The patient has given written consent for the use of their images and data in this article.
